# Gene Expression Profiles from Disease Discordant Twins Suggest Shared Antiviral Pathways and Viral Exposures among Multiple Systemic Autoimmune Diseases

**DOI:** 10.1371/journal.pone.0142486

**Published:** 2015-11-10

**Authors:** Lu Gan, Terrance P. O’Hanlon, Zhennan Lai, Rick Fannin, Melodie L. Weller, Lisa G. Rider, John A. Chiorini, Frederick W. Miller

**Affiliations:** 1 Environmental Autoimmunity Group, Clinical Research Branch, National Institute of Environmental Health Sciences, National Institutes of Health, Bethesda, Maryland, United States of America; 2 Molecular Physiology and Therapeutics Branch, National Institute of Dental and Craniofacial Research, National Institutes of Health, Bethesda, Maryland, United States of America; 3 Microarray Core, Laboratory of Toxicology and Pharmacology, National Institute of Environmental Health Sciences, National Institutes of Health, Research Triangle Park, North Carolina, United States of America; Bellvitge Biomedical Research Institute (IDIBELL), SPAIN

## Abstract

Viral agents are of interest as possible autoimmune triggers due to prior reported associations and widely studied molecular mechanisms of antiviral immune responses in autoimmunity. Here we examined new viral candidates for the initiation and/or promotion of systemic autoimmune diseases (SAID), as well as possible related signaling pathways shared in the pathogenesis of those disorders. RNA isolated from peripheral blood samples from 33 twins discordant for SAID and 33 matched, unrelated healthy controls was analyzed using a custom viral-human gene microarray. Paired comparisons were made among three study groups—probands with SAID, their unaffected twins, and matched, unrelated healthy controls—using statistical and molecular pathway analyses. Probands and unaffected twins differed significantly in the expression of 537 human genes, and 107 of those were associated with viral infections. These 537 differentially expressed human genes participate in overlapping networks of several canonical, biologic pathways relating to antiviral responses and inflammation. Moreover, certain viral genes were expressed at higher levels in probands compared to either unaffected twins or unrelated, healthy controls. Interestingly, viral gene expression levels in unaffected twins appeared intermediate between those of probands and the matched, unrelated healthy controls. Of the viruses with overexpressed viral genes, herpes simplex virus-2 (HSV-2) was the only human viral pathogen identified using four distinct oligonucleotide probes corresponding to three HSV-2 genes associated with different stages of viral infection. Although the effects from immunosuppressive therapy on viral gene expression remain unclear, this exploratory study suggests a new approach to evaluate shared viral agents and antiviral immune responses that may be involved in the development of SAID.

## Introduction

Systemic autoimmune diseases (SAID) are a group of immune disorders characterized by autoantibody production and immunopathology involving multiple mechanisms. There are several clinical phenotypes of SAID including rheumatoid arthritis (RA) and systemic lupus erythematosus (SLE) in addition to other less common phenotypes such as systemic sclerosis (SSc) and the idiopathic inflammatory myopathies (IIM). Multiple lines of evidence, including familial disease associations and disease discordance in identical twins, suggest the co-involvement of genetic and environmental factors promoting chronic inflammation and immunopathology in susceptible individuals [1–5]. While there are differences in certain risk factors, molecular pathways and the clinical expression of specific SAID phenotypes, there is a growing appreciation that in fact these diseases share a number of factors and should be studied together to best understand them [6].

The concept that infectious agents play a role in the development of autoimmune diseases has a long history [7, 8]. Many infectious agents—including viruses, bacteria and parasites—have been proposed as possible triggers of SAID. Viruses are of particular interest given their association with some autoimmune diseases based upon serologic and molecular assays, as well as evidence from certain animal models [9]. Previous studies suggested the association of many viruses, including Epstein-Barr virus [10], varicella zoster virus [11], enteroviruses [12], and hepatitis B virus [13], with the development of SAID.

Host anti-viral responses to infections may contribute to the pathophysiology of autoimmune disorders [14–16]. Among proposed mechanisms, interferon (IFN)-related pathways are the most widely studied signaling pathways involved in responses to viral infections. IFNs are a family of cytokines including type I (IFN-α, IFN-β and IFN-ω), type II (IFN-γ) and type III members, which regulate multiple biologic factors controlling inflammation and antiviral immunity [17]. The activation of the type I IFN pathway and increased expression of IFN-regulated genes are common to various SAID, including SLE [18, 19] and dermatomyositis (DM), a clinical phenotype of the IIM with characteristic skin rashes and other cutaneous findings [20, 21].

Differential gene expression analysis by microarray is ideally suited for high throughput screening of small sample quantities, and has been used successfully for viral discovery [22]. We sought to utilize twins discordant for SAID and a novel viral-human gene microarray assay using whole human genome microarray in combination with a set of over 6,000 probes, which correspond to the majority of known viral genes, to investigate the possible role of shared viral infections and the activation of shared antiviral signaling pathways in the development of SAID.

## Materials and Methods

### Study subjects

We studied 33 twin pairs discordant for SAID (14 juvenile DM, one adult polymyositis, nine juvenile idiopathic arthritis, three adult RA, one juvenile SLE, four adult SLE and one adult SSc) and 33 unrelated, healthy controls matched within five years of age, as well as by gender and ethnicity. All of these subjects were enrolled in the National Institute of Diabetes and Digestive and Kidney Diseases (NIDDK)-National Institute of Arthritis and Musculoskeletal and Skin Diseases (NIAMS) investigational review board-approved Twins-Sib study, which is evaluating relatively recent onset SAID for possible shared risk factors and pathogeneses. All adult subjects provided written informed consent, and all juvenile subjects provided written assent and their parent/guardian also provided written informed consent for their participation, as approved by the NIDDK-NIAMS institutional review board. Both adult and juvenile probands with criteria for a SAID based on American College of Rheumatology criteria, or in the case of IIM based on probable or definite Bohan and Peter criteria, were enrolled within four years of diagnosis [23]. Of the 33 twin pairs, 29 (87.9%) were monozygotic and four (12.1%) were dizygotic, and the average age and disease duration of the affected twins was 14.7± 11 and 2.1± 1.8 years, respectively. Zygosity was confirmed by an independent, commercial laboratory (Proactive Genetics, Augusta, GA). Most of the affected twins (84.8%) were being treated with immunosuppressive agents including prednisone, methotrexate, cyclosporine, mycophenolate mofetil or intravenous immunoglobulin, at the time of testing. Global disease activity and damage scores were assessed by a physician familiar with the subject on a 0–100 mm visual analogue scale and the scales from 0 to 100 indicate inactive disease/no damage to most severe disease activity/damage. There was no clinical evidence of active viral infection in any of the subjects at the time of evaluation and sample collection. Demographics, including age, gender, monozygotic/dizygotic status, and clinical information, including specific diagnosis, immunosuppressive treatment status and other clinical findings of the patients, are summarized in Table 1.

**Table 1:** Clinical characteristics of the 33 affected probands*

Clinical Characteristic	Affected probands
33 probands	15 IIM, 12 RA, 5 SLE and 1 SSc
Monozygotic/Dizygotic status	29/4
Age (years, mean± SD)	14.7± 11
Adult/Juvenile	8/25
Gender (male/female)	12/21
Disease Duration (years, mean± SD)	2.1± 1.8
Immunosuppressive Treatment	84.8% (28 of 33)
Global Disease Activity Score (0–100, mean± SD)	21.3± 17.2
Global Disease Damage Score (0–100, mean± SD)	7.8± 11.5

* IIM, idiopathic inflammatory myopathies; RA, rheumatoid arthritis; SLE, systemic lupus erythematosus; and SSc, systemic sclerosis.

### Microarray studies

RNA was extracted and purified from whole, peripheral blood samples collected in PAXgene RNA tubes (VWR Scientific, Radnor, PA, USA) using the PAXgene RNA isolation kit (Qiagen, Inc., Valencia, CA, USA) in accordance with the manufacturer’s recommendations. Purified RNA was quantified using a Nanodrop spectrophotometer (Thermo Scientific Inc., Marietta, OH, USA) and stored at -80°C until further analysis. Gene expression analysis was performed with a custom DNA microarray using the Agilent 4 x 44 K whole human genome microarrays that include 44,000 probes for nearly 20,000 human genes (Agilent Technologies, Inc., Santa Clara, CA, USA) with the addition of over 6,000 oligo probes for known viral genes. The custom microarray design and data validation were described earlier [24]. To identify viruses in clinical samples, we developed a computational method that identifies conserved viral sequences in a specific viral open reading frame. If sufficient annotation existed, multiple open reading frames were chosen both in early and late transcription regions. Using this approach we obtained the viral sequence data from the well-established database of viral genomes in GenBank and developed a set of over 6,000 probes that covered 54 virus families. RNA quality was verified using a 2100 Bioanalyzer (Agilent Technologies, Palo Alto, CA) and only RNA samples with RIN score > 7 were used for the following arrays. A total of 500ng RNA was amplified and labeled using the Agilent fluorescent linear amplification kit following the manufacturer’s standard protocol. For each array, 750 ng of each Cy5- (human universal control) and Cy3-labeled sample cRNA were hybridized using the Agilent *in situ* hybridization kit. Hybridizations were performed for 17 hours in a rotating hybridization oven. Slides were washed and then scanned with an Agilent scanner. Data were then obtained using Agilent Feature Extraction software (Agilent Technologies, Inc., Santa Clara, CA, USA), which performed error modeling and adjusting for additive and multiplicative noise. The microarray data used in this publication have been deposited in NCBI gene expression and hybridization array data repository: Gene Expression Omnibus (GEO) and are accessible through series accession number [GSE74027].

### Microarray data analysis

GeneSpring GX 12.6 (Agilent Technologies, Palo Alto, CA) was utilized to filter and normalize the raw data. Quantile normalization and a baseline transformation to all medians of samples were used. Paired *t* tests were used to identify oligo probes differentially expressed among affected probands (P), their unaffected twins (U) and the matched, unrelated healthy controls (C). We performed three family-based pair comparisons using whole human genome probes and custom viral probes separately: (1) P vs. C, in which all probands were compared with matched, unrelated healthy controls; (2) U vs. C, in which all disease unaffected twins were compared with matched, unrelated healthy controls; (3) P vs. U, in which all probands were compared with their unaffected twins. Stoney’s *q*-value was used to adjust *p* values for multiple comparisons and a *q*-value cutoff of 0.05 was selected to show differential gene expression with statistical significance [6]. Additionally, Pearson’s correlation coefficients were calculated to evaluate the association between HSV-2 expression and the use of immunosuppressive therapy.

### Ingenuity pathway analysis

Human genes, which were significantly differentially expressed between probands and their unaffected twins, were analyzed using QIAGEN’s Ingenuity^®^ Pathway Analysis platform (IPA^®^, QIAGEN Redwood City, www.qiagen.com/ingenuity). Each gene of interest was mapped onto a global molecular network derived from information contained in the IPA knowledge base. Those genes that were overexpressed in the dataset were divided into disease and biological function subgroups and those representing canonical pathways were determined. The number of probes mapped to each biologic pathway that differed from the number expected due to chance alone was assessed by Fisher’s exact test.

### Real-time quantitative polymerase chain reaction (qPCR) and quantitative reverse-transcription PCR (qRT-PCR)

Relative quantitation (RQ) measurements of HSV-2 gene targets were performed using qPCR with subjects’ DNA as source material. qRT-PCR was performed using subjects’ reverse transcribed RNA samples purified from whole, peripheral blood collected from disease-discordant twins and unrelated, matched controls. Briefly, cDNA was prepared using the High Capacity cDNA Reverse Transcription kit (500 μg of input RNA per subject) as specified in the manufacturer’s protocol (Applied Biosystems, Foster City, CA, USA). qPCR and qRT-PCR assays were performed in triplicate using custom primers. Standard SYBR^®^ Green assays (20 μL) were performed using a Power SYBR^®^ Green Master Mix and a ViiA^™^ 7 Real-Time PCR system following the manufacturer’s standard protocol (Life Technologies, Durham, NC, USA). Co-amplification of the housekeeping gene human glyceraldehyde-3-phosphate dehydrogenase (GAPDH) served as an endogenous, standardization control. The relative expression of each gene was calculated from log-phase mean threshold cycle values normalized to GAPDH and calculated as reported previously [23].

## Results

### Microarray profiling of human gene expression

**Fig 1:**
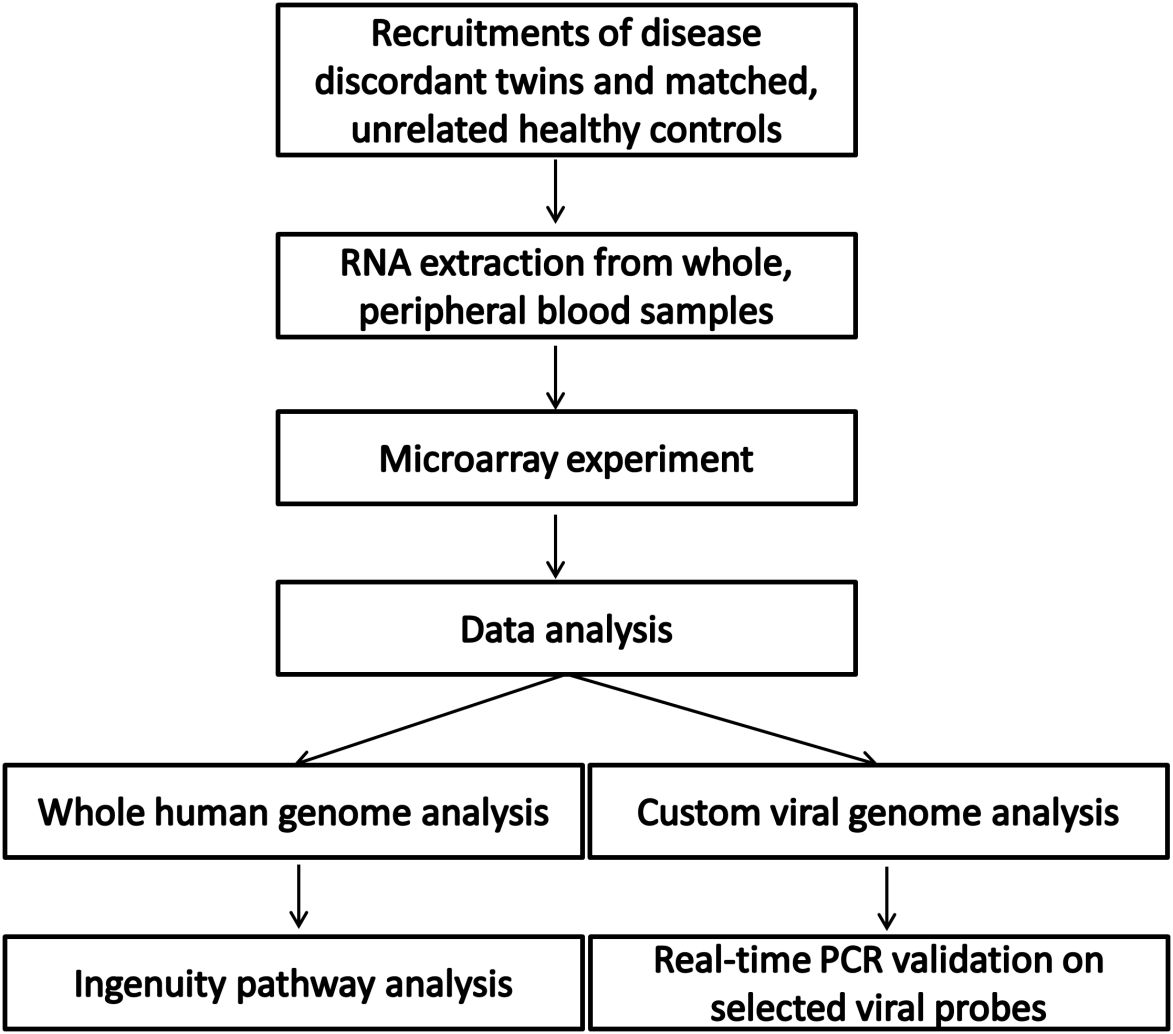
Flow chart of the experimental design and data analyses.

A flow chart of the experimental work and data analysis plan is shown in Fig 1. A comparison of human gene expression between SAID and their unaffected twins revealed significant differences in 789 human oligo probes (corresponding to 537 human genes, S1 Table). Similarly, 1,666 human oligo probes (corresponding to 1,041 human genes, S1 Table) differed in comparisons of gene expression between probands and unrelated, healthy controls. No significant differences in human gene expression were detected in similar comparison between disease unaffected twins and unrelated, healthy controls. Probe sets that are common to the first two comparisons (P vs. U and P vs. C) are represented in S1 Fig. We next focused our analysis on the differential human gene expression detected between the SAID discordant twins (789 human oligo probes) in an effort to minimize the influence of natural genetic variation between the comparison groups.

### Biologic pathway analysis

**Fig 2:**
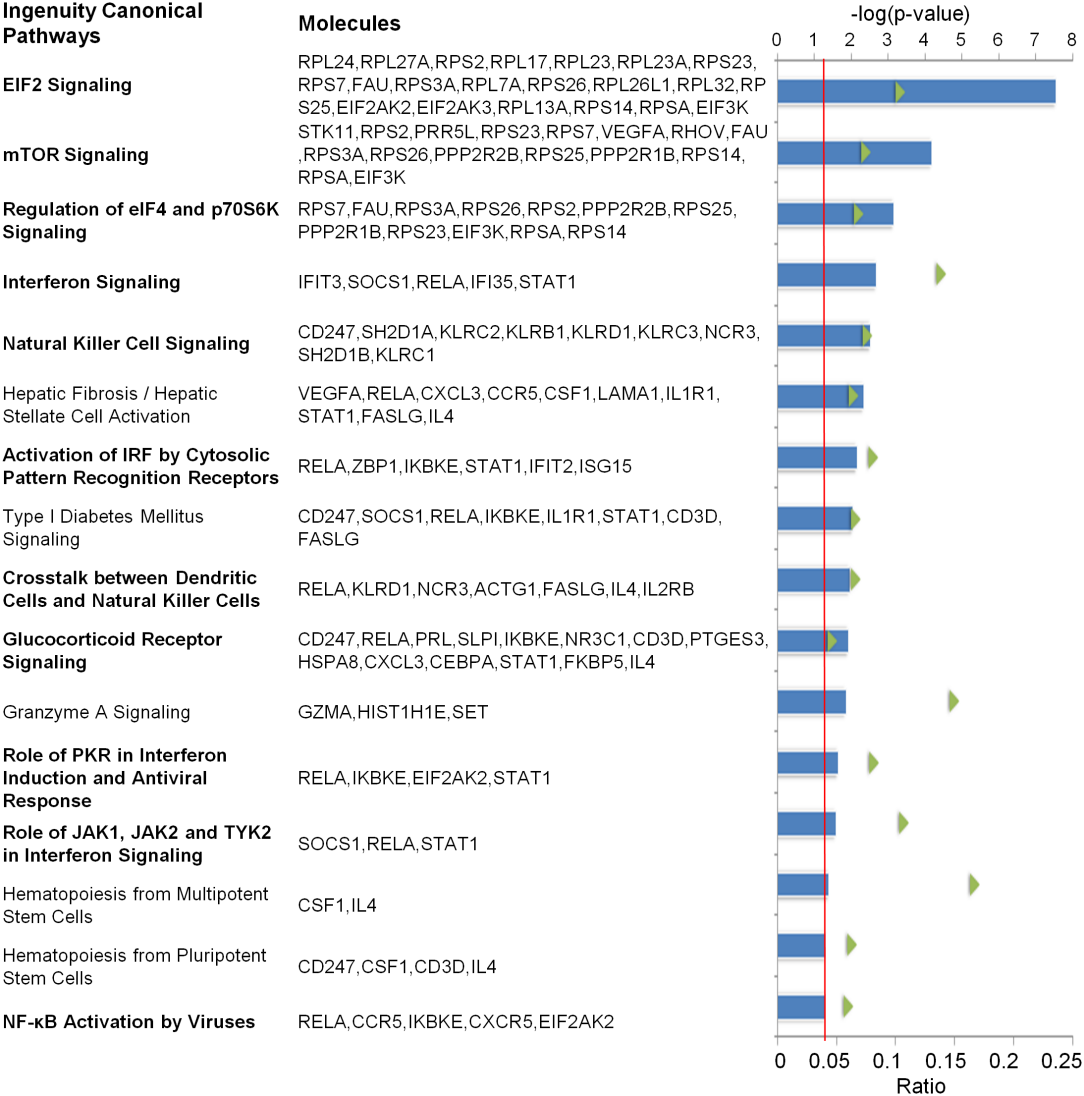
Ingenuity pathway analysis of differentially expressed human genes between disease discordant twins. The analysis compared 789 oligo probes corresponding to 537 human genes significantly differentially expressed (fold change >1.5, *q* <0.05) between twins discordant for SAID. The upper horizontal axis (blue bars) describe the association of the data set with a given pathway (-log (*p* value)). The cutoff threshold value (defined as *p* = 0.05) is shown by the vertical red line. The ratio of the number of genes from the data set that map to a given pathway divided by the total number of molecules that comprise the pathway is shown on the horizontal axis (green triangles). Previously reported viral-related signaling pathways are in bold print.

In a smaller, previous study, our group reported differential human gene expression between 20 SAID probands and unrelated, healthy controls. Many of the differentially expressed genes mapped to shared cell signaling pathways associated with immune-regulation and inflammatory responses [23]. To further assess these findings, we performed IPA on our larger current dataset to determine whether significantly differentially expressed genes might be associated with common shared biologic pathways in SAID. As shown in Fig 2, statistically significant differences between affected and unaffected twins included genes associated with several canonical biologic pathways involved in multiple, overlapping viral signaling functions. Among these, the activation of IFNs and several IFN-related signaling pathways, including the activation of interferon regulatory factors (IRF) by cytosolic pattern recognition receptors, protein kinase R (PKR) in interferon induction and antiviral responses, the Janus kinases (JAK1, JAK2) and tyrosine kinase 2 (TYK2), were prominent. Other viral infection-related signaling pathways included eukaryotic initiation factor 2 (eIF2), mammalian target of rapamycin (mTOR), eukaryotic initiation factor 4 (eIF4)/ p70S6K, glucocorticoid receptor, NF-kappa B and innate cell-related pathways (natural killer cells and dendritic cells).

Among the 537 significantly differentially expressed human genes between SAID discordant twins, 330 and 207 were up- and down-regulated, respectively. We next examined the disease and functional clustering of these genes. The top five diseases and disorders in the dataset were infectious diseases (*p* values range = 6.71 x 10^−9^ to 6.82 x 10^−3^, 127 molecules), inflammatory responses (3.24 x 10^−12^ to 6.78x 10^−3^, 117 molecules), inflammatory disease/disorders (2.44 x 10^−7^ to 5.36 x 10^−3^, 100 molecules), immunologic disease/disorders (5.25 x 10^−9^ to 6.53 x 10^−3^, 91 molecules), and connective tissue disorders (2.44 x 10^−7^ to 4.94 x 10^−3^, 78 genes). Of the 127 infectious disease-related genes differentially expressed between disease discordant twins, 107 were viral-related. INTERFEROME, a database of interferon regulated genes [24], was also used to confirm the identity of IFN-regulated genes (IRGs). Forty-one IRGs (38.3%, 41/107) were differentially expressed in the peripheral blood RNA from probands compared to their unaffected twins, including 28 (68.3%, 28/41) up-regulated and 13 (31.7%, 13/41) down-regulated genes (Table 2). Consistent with a previous study [25], a subset of seven overexpressed genes (*RSAD2*, *IFI44*, *ISG15*, *OAS3*, *EPSTI1*, *IFIT3*, *IFI6*) comprising an IFN signature were detected in the peripheral blood of SAID probands; four of these (*RSAD2*, *IFIT3*, *IFI6*, *ISG15*) were viral-related (Table 2).

**Table 2:** IFN-regulated genes that were significantly differentially expressed between SAID probands and their unaffected twins*

Gene Symbol	Entrez Gene Name	Fold Change	*q* value
**Increased expression in probands**			
*LTF*	lactotransferrin	2.7	0.02
*RSAD2*	radical S-adenosyl methionine domain containing 2	2.4	0.05
*IFIT3*	interferon-induced protein with tetratricopeptide repeats 3	2	0.03
*STAT1*	signal transducer and activator of transcription 1, 91kDa	2	0.03
*IFI6*	interferon, alpha-inducible protein 6	1.9	0.03
*SOCS1*	suppressor of cytokine signaling 1	1.9	0.01
*ISG15*	ISG15 ubiquitin-like modifier	1.9	0.05
*OASL*	2'-5'-oligoadenylate synthetase-like	1.8	0.04
*IFIT2*	interferon-induced protein with tetratricopeptide repeats 2	1.8	0.02
*DDX60L*	DEAD (Asp-Glu-Ala-Asp) box polypeptide 60-like	1.7	0.01
*VEGFA*	vascular endothelial growth factor A	1.7	0.01
*HP*	haptoglobin	1.7	0.03
*CD274*	CD274 molecule	1.7	0.02
*ZBP1*	Z-DNA binding protein 1	1.6	0.03
*S100A12*	S100 calcium binding protein A12	1.6	0.05
*SAMD9*	sterile alpha motif domain containing 9	1.6	0.01
*DYSF*	dysferlin	1.6	0.04
*RELA*	v-rel avian reticuloendotheliosis viral oncogene homolog A	1.6	0.03
*CSF1*	colony stimulating factor 1 (macrophage)	1.6	0.02
*ACSL1*	acyl-CoA synthetase long-chain family member 1	1.6	0.05
*GH2*	growth hormone 2	1.6	0.04
*APOBEC3B*	apolipoprotein B mRNA editing enzyme, catalytic polypeptide-like 3B	1.6	0.01
*SPP1*	secreted phosphoprotein 1	1.6	0.03
*CXCL3*	chemokine (C-X-C motif) ligand 3	1.6	0.03
*TRIM25*	tripartite motif containing 25	1.6	0.01
*EIF2AK2*	eukaryotic translation initiation factor 2-alpha kinase 2	1.5	0.03
*MYC*	v-myc avian myelocytomatosis viral oncogene homolog	1.5	0.03
*IFI35*	interferon-induced protein 35	1.5	0.01
**Decreased expression in probands**			
*TRERF1*	transcriptional regulating factor 1	-2.1	0.01
*TMED2*	transmembrane emp24 domain trafficking protein 2	-2.1	0.01
*SNRPF*	small nuclear ribonucleoprotein polypeptide F	-2	0.01
*HLA-DQB1*	major histocompatibility complex, class II, DQ beta 1	-1.9	0
*DDX6*	DEAD (Asp-Glu-Ala-Asp) box helicase 6	-1.9	0.02
*CCR5*	chemokine (C-C motif) receptor 5 (gene/pseudogene)	-1.7	0.01
*TOX*	thymocyte selection-associated high mobility group box	-1.6	0.03
*MAGT1*	magnesium transporter 1	-1.6	0.01
*IL2RB*	interleukin 2 receptor, beta	-1.6	0.01
*GZMA*	granzyme A (granzyme 1, cytotoxic T-lymphocyte-associated serine esterase 3)	-1.6	0.02
*SH2D1A*	SH2 domain containing 1A	-1.6	0.01
*CD200R1*	CD200 receptor 1	-1.5	0.02
*CMKLR1*	chemokine-like receptor 1	-1.5	0.04

*Listed are the 41 IFN-regulated genes of 107 viral infection-related genes identified by the INTERFEROME program that were statistically significant (*q* < 0.05) and had a fold change >1.5 between disease discordant twins. Fold change values indicate increase (positive) or decreased (negative) levels of gene expression in probands related to unaffected twins.

### Microarray screening for differential viral gene expression

A cluster analysis of viral oligo probes for the three study groups demonstrated a clear trend of viral gene up-regulation in the peripheral blood of SAID probands compared to either unaffected twins or healthy controls. However, data from both human and viral gene microarrays demonstrated that unaffected twins clustered to a greater extent with unrelated, healthy controls than their SAID affected twins. Significant differences in peripheral blood viral gene expression were observed between probands and both the unaffected twins and unrelated, healthy controls while no significant differences in viral gene expression were observed between unaffected twins and unrelated, healthy controls. A total of 64 viral probes (from 40 different viruses) distinguished SAID probands from the unaffected twins and healthy control groups (S1 Fig). Among these viruses, HSV-2 is the only known human pathogen and was verified using four distinct oligo probes corresponding to three different HSV-2 genes. *UL19* (encoding the major capsid protein), *UL36* (encoding a tegument protein), and *RS1* (encoding infected cell polypeptide 4, the major transcriptional activator for progression beyond the immediate-early phase of viral infection) are the three different HSV-2 genes with different functions in the viral infection cycle (Table 3). Of interest, a variety of viruses, including Epstein-Barr virus (EBV), parvovirus B19, and varicella zoster virus, that have been previously suggested to be related to the pathogenesis of SAID and were included in the viral array, were not found to be differentially expressed in individuals affected with SAID. Subgroup analyses selected for monozygosity, pediatric age, RA, and IIM demonstrated similar patterns of differential viral gene expression. Significant differences in viral gene expression were not detected in the SLE and SSc subgroups and this is likely attributable to their smaller sample sizes (data not shown). We used principal component analysis (PCA) to evaluate the effect of clinical variables, such as the presence of immunosuppressive therapy (S2 Fig), the duration of disease, physician global disease activity scores or disease damage levels and observed no differences across the first three principal components due to these variables (S2 Fig) [23].

**Table 3:** Four distinct viral oligo probes from three HSV-2 genes that were differentially expressed between probands and unaffected twins, as well as between probands and matched, unrelated healthy controls*.

Probe	Gene Symbol	Genecategory	Protein	Function	FC P/U(*q* value)	FC P/C(*q* value)	Probe Sequence
1	*UL19*	Late Gene	Major capsid protein	Capsid morphogenesis	2.1 (0.011)	2.0 (0.016)	GCGCGGCACGGCCGACCAGATGCTGCACGTGCTGTTGGAGAAGGCGCCTCCCCTGGCCCT
2	*UL19*	Late Gene	Major capsid protein	Capsid morphogenesis	1.9 (0.023)	2.5 (0.005)	CCAGATGCTGCACGTGCTGTTGGAGAAGGCGCCTCCCCTGGCCCTGCTGTTGCCCATGCA
3	*UL36*	Late Gene	Very large tegument protein	Capsid transport	1.6 (0.048)	2.5 (0.001)	GCCCTGGGCCCCGAGGCCATCCAGGCGCGGCTGGAGGACGTGCGGATCCAGGCCCGCCGG
4	*RS1*	Immediate Early Gene	Transcriptional regulator ICP4	Gene regulation	1.7 (0.03)	3.4 (0.001)	CATCGCGACCTCGGCCCCGCGGCCCTGCGTCGTCGTCGTCGTCTTCTTCTTCTTCCGCTG

*Paired comparisons were made between probands (P) and unaffected twins (U) or matched, unrelated healthy controls (C). Statistically significant viral probes (fold change, FC >1.5, *q* <0.05) were selected.

### HSV-2 viral gene analyses

**Fig 3:**
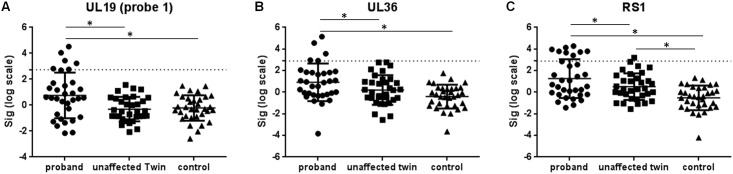
Dot plots of three HSV-2 gene expression levels in SAID affected probands, unaffected twins, and matched, unrelated healthy controls. The individual normalized signal data for *UL19* (A, probe 1 in Table 3), *UL36* (B) and *RS1* (C) were plotted according to the three study groups: probands, unaffected twins, and matched, unrelated healthy controls. Statistically significant *p* values between any two groups are shown (*) and were calculated using one-way ANOVA with corrections for multiple comparisons. The proposed “normal range” values were derived from the mean plus three standard deviations (SD, dash line) of healthy control expression values.

In total, four HSV-2 viral gene probes were detected with significant increased viral gene expression in SAID probands compared to either unaffected twins or unrelated, healthy controls (Table 3). Dot plots of viral gene expression levels for three different viral probes (corresponding to three different HSV-2 genes) revealed consistent overexpression of these HSV-2 genes in the peripheral blood from some individual probands compared to their unaffected twins and unrelated, healthy controls (Fig 3A–3C). The peripheral blood from six probands was positive for *UL19*, while five probands were positive for *UL36* and eight were *RS1* positive. Five probands were positive for two different HSV-2 genes while two probands were positive for three different HSV-2 genes. HSV-2 gene expression was also detected among different disease phenotypes, including two RA, two SLE, and five IIM patients. Additionally, we did not find any correlations between the use of immunosuppressive therapy and the levels of HSV-2 gene expression (*UL19*, r = -0.042, *p* = 0.814; *UL36*, r = 0.032, *p* = 0.868; *RS1*, r = 0.042, *p* = 0.817), which suggests that the three HSV-2 gene expressions were not modulated by immunosuppressive therapy.

### qPCR and qRT-PCR validation

Relative quantification of differential viral gene expression among SAID probands and matched, unrelated healthy controls was independently evaluated by qPCR and qRT-PCR for two HSV-2 genes (*UL36* and *RS1*) as described previously (see Materials and methods). As summarized in Table 4, both HSV-2 genes evaluated by either qPCR or qRT-PCR showed the same trends of increased viral gene detection and expression as those observed from the microarray analysis in comparisons of probands with matched, unrelated healthy controls.

**Table 4:** Comparisons of differential gene expression values determined by relative quantitative-polymerase chain reaction and microarray analyses*.

HSV-2 genes	Microarray FC* P/C (All)	qPCRFC P/C (All)	qPCRFC P/C (Positive)	qPCRFC P/C (Negative)	qRT-PCR FC P/C (All)	qRT-PCR FC P/C (Positive)	qRT-PCR FC P/C (Negative)	Primers
*UL36*	2.5	1.5	2.3	0.8	1.3	1.5	1.0	For: TCTGCGTCGGAGTGTTCATCRev: GACTTCGTGGGCTTCCTCTC
*RS1*	3.4	1.3	1.7	0.9	1.2	1.4	1.0	For: GATGAAGGAGCTGCTGTTGCRev: GATGGGGTGGCTCCAGAAC

* Fold change (FC) values indicating relative expression levels of two HSV-2 genes (*UL36* and *RS1*) between two study groups: probands (P) and matched, unrelated healthy controls (C). qPCR, quantitative polymerase chain reaction; qRT-PCR, quantitative reverse-transcription PCR; Positive, the affected probands with probe values above the proposed normal range (mean plus three standard deviation of control group); Negative, the affected probands with probe values within the proposed normal range.

## Discussion

Several mechanisms have been proposed to explain how viral infections might trigger the onset of autoimmune disorders [17, 26]. One example is molecular mimicry, wherein a viral protein(s) shares similar structures with self-antigens resulting in a break of immunological tolerance [27]. Viral infections might also activate the immune system through pattern recognition receptors activating downstream anti-viral signaling pathways that culminate in inflammatory and possibly autoimmune responses. Pro-inflammatory signaling pathways include IRF and NF-kappa B activation which result in the production of IFNs and regulatory cytokines. Several signaling molecules identified in our study and others (e.g. JAK1, JAK2 and TYK2) are also involved in interferon gene activation. During the antiviral response, protein kinase R (PKR), an interferon-induced enzyme, is known to activate eIF2 to inhibit viral mRNA translation and viral protein synthesis. In the early phases of antiviral responses, innate immune cells, *e*.*g*., natural killer cells [28] may cooperate with dendritic cells [29] to counteract viral evasion mechanisms. Although it is known that there are many clinical, immune and molecular differences among the different SAID phenotypes, our study was focused on identifying signaling pathways and viral gene expressions that are shared among SAID. Our data corroborate previous studies that in fact there are many shared pathways, with an emphasis on IFN signature, and at least one shared viral gene expression pattern relating to HSV [30].

Despite the fact that viral infection has been associated with the disease pathology of SAID (*e*.*g*., IIM [8], SLE [31], RA [32], and SSc [33]), there is little evidence for viruses acting as direct triggers for SAID onset. Following primary infection, certain pathogenic viruses remain clinically latent in the host until reactivated possibly years or decades later. The reactivation of pathogenic viruses is also a recognized complication of immunosuppressive therapy. Because the majority of affected probands with SAID in our study were treated with immunosuppressive agents at the time of testing, it is difficult to distinguish between the timing of primary viral infection, disease onset, and possible viral reactivation after immunosuppressive therapy. However, we did not detect any statistical co-variations between SAID affected twins having measurable HSV-2 gene expression and the type or dosage of immunosuppressive therapies, disease duration, physician global disease activity scores or disease damage. The lack of any correlation of immunosuppressive therapy, as well as the duration, extent or severity of disease activity, with the level of viral gene expression suggests that viral gene expression was not modulated by immunosuppression or disease processes after disease development. Moreover, while the expression of certain genes may be altered by glucocorticoid treatments [34], these do not include the majority of genes in the anti-viral pathways identified in this study. Therefore, we suggest that the HSV-2 signals are bio-relevant due to a lack of correlations or association trends observed between those subjects with active HSV-2 gene expression and the presence or type of immunosuppressive therapy, disease duration, physician global disease activity scores or disease damage levels.

Surprisingly, in our study, HSV-2 was the only human viral pathogen identified in the peripheral blood RNA and DNA of SAID affected twins by viral microarray and confirmatory, quantitative, targeted gene PCR analyses (*UL36* and *RS1*). Interestingly, while previous studies linked other herpes viruses such as EBV with human autoimmunity [35], we did not find increased EBV gene expression, which was assessed by *UL19* (encoding the major capsid protein) and *UL36* (encoding a tegument protein) genes, in SAID probands. Although cases of HSV-2 infection have been reported in patients with pemphigus vulgaris [36], autoimmune thyroid disease [37], or DM [38], a convincing role for HSV-2 infection in the etiology of SAID has not been established. Previous findings have also demonstrated early infections with HSV-2, including maternal infection, as being possibly associated with schizophrenia [39]. The prevalence of HSV-2 infection increases with age and peak incidence occurs in 15–24 year olds. The average age of SAID affected twins in our study was 14.7 ± 11 years.

In general, HSV-2 genes are classified into three groups: immediate early (IE), early (E) and late (L) genes according to their biologic functions. IE genes, including five members (*RL1*, *UL54*, *RS1*, *US1* and *US12*), are transcribed immediately after viral infection. E genes, including at least 12 genes, are involved in HSV-2 DNA replication. The remaining L genes are virion components and include the capsid (*UL19*), tegument (*UL36*) and envelope proteins. Our report of increased IE gene (*RS1*) and L gene (*UL19*, *UL36*) expression suggest that HSV-2 may actively replicate in SAID affected twins and suggests a possible role for HSV-2 viral infection in some patients, possibly maintaining or enhancing inflammatory processes.

We reported previously that human genes differentially expressed between 20 SAID discordant twin pairs and unrelated, healthy controls were associated with multiple immunoregulatory and inflammatory pathways among probands [23]. The present study is an expanded analysis of 33 SAID discordant twin pairs and 33 matched, unrelated healthy controls. In addition, we have used a novel, custom microarray to measure differential viral and human gene expression in concert. Our study did have several limitations, including small sample sizes of individual SAID phenotypes, heterogeneity of SAID phenotypes, the lack of gene expression data in different cell subsets and the use of immunosuppressive therapies among probands. These limitations result in part from the rarity of qualified SAID-discordant twin pairs identified for the study. The disease discordant twin study design was selected to help minimize the effects of natural genetic variations among human subjects in clinical studies. Additionally, we performed subgroup analyses and found no difference by disease phenotype, which supports the hypothesis that different SAID may share certain etiopathogenic mechanisms including those of viral origin. However, some subgroup analyses were likely to be underpowered due to the small sample sizes to evaluate the effect of the clinical variables (*i*.*e*. the dose of any particular immunosuppressive agent).

In conclusion, the increased and shared expression of certain human antiviral pathways and HSV-2 viral genes among multiple SAID disease discordant twins suggest a possible role for HSV-2 in some patients with SAID. Future studies are required to more fully assess the etiologic role of viral infection (*e*.*g*., herpes- and other viruses) in the development of human SAID. The frequent detection of type I IFN signatures reported among our and other populations of SAID patients suggests that perturbations in the transcriptional control of multiple IFN-regulated genes may be a consequence of viral infection or reactivation in some susceptible individuals.

## Supporting Information

S1 Fig Gene expression analyses using microarray. Venn diagrams by Gene Spring represent the human (A) and viral (B) gene probes that were significantly (*q* <0.05) differentially expressed between probands and their unaffected twins or unrelated, healthy controls.
(TIF)

S2 Fig Principal component analysis of the effects of immunosuppressive therapy in probands. The analysis includes all viral oligo probes used in this study. PC, principal component; 0, No immunosuppressive therapy; 1, one immunosuppressive agent; 2+, two or more immunosuppressive agents.
(TIF)

S1 Table Differential human gene expression in paired comparisons. (XLS)
