# Supplementary figures and images for: Gene Expression Profiles from Disease Discordant Twins Suggest Shared Antiviral Pathways and Viral Exposures among Multiple Systemic Autoimmune Diseases
Source: PLoS One. 2015 Nov 10;10(11):e0142486. doi: 10.1371/journal.pone.0142486 (PMC4640563; doi:10.1371/journal.pone.0142486)

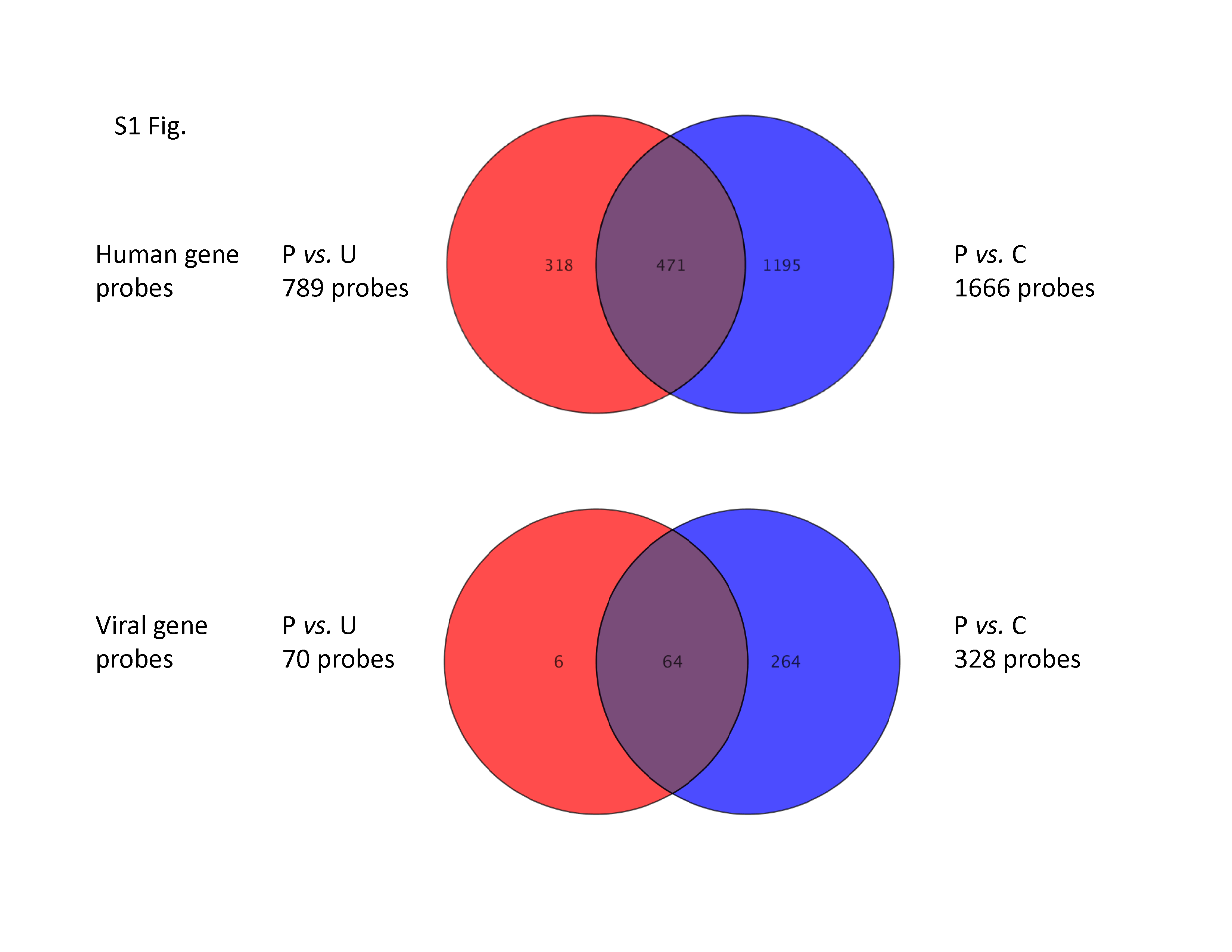

Supplement: S1 Fig — Venn diagrams by Gene Spring represent the human (A) and viral (B) gene probes that were significantly (q <0.05) differentially expressed between probands and their unaffected twins or unrelated, healthy controls. (TIF) [file pone.0142486.s001.tif]

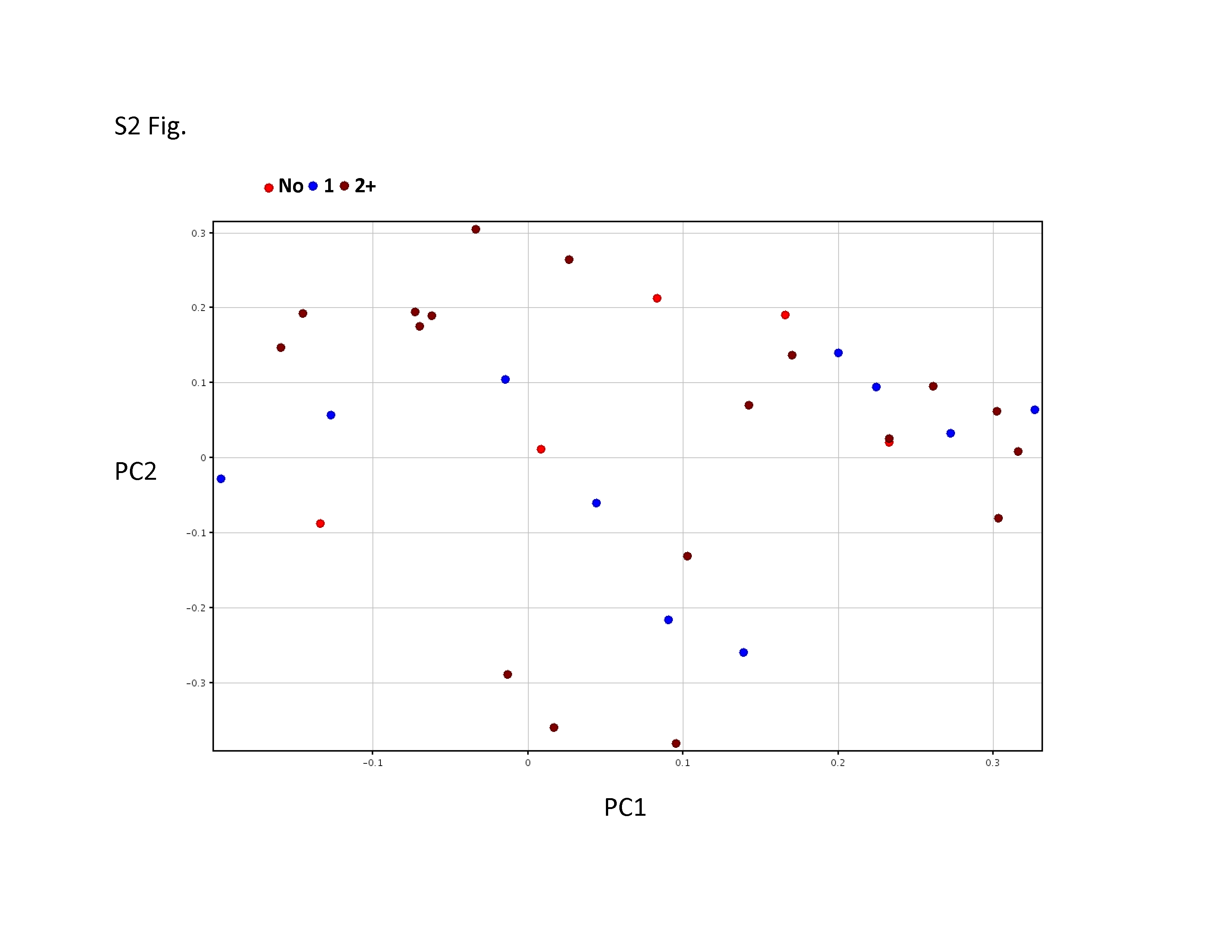

Supplement: S2 Fig — The analysis includes all viral oligo probes used in this study. PC, principal component; 0, No immunosuppressive therapy; 1, one immunosuppressive agent; 2+, two or more immunosuppressive agents. (TIF) [file pone.0142486.s002.tif]
